# Central venous catheters do not increase the hemorrhagic risk in acute promyelocytic leukemia patients during induction therapy

**DOI:** 10.3389/fonc.2024.1332372

**Published:** 2024-04-12

**Authors:** Manxiong Cao, Jiaqiong Hong, Dongqing Zhang, Feiheng Chen, Yongzhong Su

**Affiliations:** ^1^ Department of Hematology, The First Affiliated Hospital of Shantou University Medical College, Shantou, Guangdong, China; ^2^ Department of Laboratory Medicine, The First Affiliated Hospital of Shantou University Medical College, Shantou, Guangdong, China

**Keywords:** central venous catheter, hemorrhage, coagulopathy, acute promyelocytic leukemia, induction therapy

## Abstract

In acute promyelocytic leukemia (APL), hemorrhage, particularly intracranial hemorrhage, is the most common cause of early death. A central venous catheter (CVC) may provide a greater guarantee of safety and comfort to APL patients. However, CVCs have seldom been attempted in APL patients during induction therapy because of concerns about increasing the risk of hemorrhagic complications after this invasive procedure. To evaluate the hemorrhagic risk after CVC placement in APL patients during induction therapy, we retrospectively analyzed 95 newly diagnosed patients with APL from January 2010 to December 2022. Among these patients, 39 patients in the CVC group and 56 patients in the non-CVC group were included. Laboratory and clinical parameters of the two groups were collected and compared. There were no significant differences in median platelet, fibrinogen (Fbg), D-dimer, prothrombin time (PT), white blood count (WBC) and hemoglobin (Hb) between the CVC and non-CVC groups on the first day of the visit (day 0) and the following days (day 4, day 7, day 11, day 14, day 18 and day 21) (*p* = 0.382, *p* = 0.805, *p* = 0.456, *p* = 0.902, *p* = 0.901 and *p* = 0.097, respectively). The consumption of transfused platelets and Fbg was not significantly different between the CVC group and non-CVC group (5.0 vs. 4.5 units, *p* = 0.34, and 6.8 vs. 6.0, *p* = 0.36, respectively). The last day of platelet and Fbg transfusion was also not significantly different (21 vs. 19, *p* = 0.238 and 7.5 vs. 8.5, *p* = 0.684, respectively). The incidences of total hemorrhagic events and hemorrhagic death were lower in the CVC group than in the non-CVC group (17.9% vs. 37.5%, *p* = 0.04 and 0% vs. 16.1%, *p* = 0.01, respectively). The 30-day survival rate was not significantly different (92.3% vs. 82.1%, respectively, *p* = 0.145) for the CVC group compared with the non-CVC group. Our study suggested that CVCs did not increase the hemorrhagic risk in APL patients during induction therapy and that a CVC should be considered in this type of clinical situation.

## Introduction

A high cure rate above 90% of APL patients with treatment that combines all-trans retinoic acid (ATRA), arsenic compounds and anthracycline-based chemotherapy has been demonstrated by several groups (1–3). Nevertheless, death within the first 30 days after diagnosis remains the most common cause of treatment failure (4). Hemorrhage, particularly intracranial hemorrhage, is the most common cause of early death (5, 6). The coagulation disorder in APL results from the combination of disseminated intravascular coagulation (DIC), fibrinolysis, proteolysis, thrombocytopenia, and platelet dysfunction (7).

CVCs provide a greater guarantee of safety and comfort during chemotherapy to cancer patients because many chemotherapies are infused through an intravenous access and may damage peripheral blood vessels (8), and repeated venipuncture is an unpleasant experience for patients (9). However, CVC insertion has seldom been attempted in APL patients during induction therapy because of concerns about increasing the risk of hemorrhagic complications after this invasive procedure, leading to guidelines such as those from an expert panel of the European LeukemiaNet that do not recommend the routine use of CVCs (10–12). Thus, few studies have attempted CVCs in APL patients during induction therapy. Our institution had inserted CVCs in some patients with APL, and more than ten years of data were available for this study. The aim of this retrospective study was to evaluate the hemorrhagic risk after CVC in APL patients during induction therapy by comparing the laboratory and clinical parameters between the CVC and non-CVC groups.

## Patients and methods

### Patients

All patients ≥14 years of age with newly diagnosed APL at the First Affiliated Hospital of Shantou University Medical College between January 2010 and December 2022 were included in this retrospective, single-center data analysis. This study was approved by the First Affiliated Hospital of Shantou University Medical College Ethics Committee and conducted in accordance with the Declaration of Helsinki.

The APL diagnosis was based on morphology and immunophenotyping and confirmed by the presence of t (15; 17) translocation by karyotyping, immunofluorescence *in situ* hybridization or a real-time polymerase chain reaction for the PML/RARα transcript on bone marrow samples. During the study period, 121 patients were enrolled in our center. Among those, the following patients were excluded: 9 patients who were <14 years old, 11 patients transferred to other hospitals or refused to receive treatment, and 6 patients who relapsed or were not newly diagnosed. In the current analysis, a total of 95 hospitalized patients were included.

Based on the use of a CVC and the coagulopathies that resolved within 14 days in most patients (13), the patients were divided into two groups: the CVC group (CVC inserted ≤14 days from admission) and the non-CVC group (CVC not inserted or inserted beyond 14 days from admission). There were 39 and 56 patients in the CVC and non-CVC groups, respectively. Furthermore, when we analyzed the dynamic change in hemostatic variables, 13 early death cases were excluded to minimize interference as much as possible.

Based on the induction regimens, patients were divided into four groups: the all-trans retinoic acid (ATRA) with arsenic trioxide (ATO) group, ATRA with chemotherapy group, ATRA and ATO with chemotherapy group, and ATRA monotherapy group. To overcome coagulopathy, platelet transfusions were given to maintain a platelet count of more than 30 × 10^9^/L until resolution of the coagulopathy. Once the patient’s coagulopathy was under control, platelet transfusions were only given for patients with hemorrhagic manifestations or when the platelet count dropped below 20 × 10^9^/L. Fbg, cryoprecipitate and/or fresh-frozen plasma was transfused to maintain Fbg >150 mg/dl. Heparin prophylaxis was not recommended, except for the treatment of thrombotic events. One unit of cryoprecipitate or 100 ml of plasma was converted to 0.25 g of Fbg in favor of the subsequent statistical analysis.

### CVC insertion and management

Decisions related to directing APL patients to position the CVCs were entirely made by the treating physician mainly for chemotherapy, monitoring hemodynamics and transfusion. PICCs were used when the duration of systemic anticancer therapy was 3 months or longer or there were no adequate peripheral veins. Centrally inserted central catheters (CICCs) were used when critically APL patients required hemodynamic monitoring and transfusion for a treatment duration of 2–3 weeks.

All CVCs were inserted by various specialists such as nurse practitioners. Before the puncture, an ultrasound examination is performed to assess the anatomy of the target vein and adjacent anatomic structures. After understanding the positional relationships of anatomical landmarks on the body surface, the anatomical landmark methods were used to position the catheter and when possible, ultrasound-guided methods were used to minimize bleeding complication rates. At the end of the procedure, a chest radiograph was routinely performed to determine if the catheter tip location was placed at the cavoatrial junction. Regular device checks took place according to the protocol of our institution. Insertion-site care entailed weekly changes of transparent, semi-permeable film-covered dressing.

### Laboratory data

To verify an association between CVC and recovery from coagulopathy, data from day 0 to day 21 were collected for patients who survived the first 30 days. The main laboratory data were collected from the patients’ hospital files. The dynamic changes in WBC, hemoglobin (Hb) and coagulation indexes, including platelets, PT, activated partial thromboplastin time (APTT), D-dimer, and Fbg, were collected on the first day of the visit (day 0) and after induction treatment (days 4, 7, 11, 14, 18, and 21). The percentages of promyelocytes and blasts in the bone marrow and peripheral blood were determined by microscopic examinations by two experienced physicians independently. Disseminated intravascular coagulation (DIC) was defined under the criteria for the DIC scoring system released by the International Society of Thrombosis and Hemostasis (ISTH) (14). The risk stratification of APL is based on WBC and platelets: WBC of ≤10 × 10^9^/L and platelets >40 × 10^9^/L as low risk, WBC ≤10 × 10^9^/L and platelets <40 × 10^9^/L as intermediate risk, and WBC >10 × 10^9^/L as high risk.

### Follow-up of hemorrhagic and thrombotic events

To verify an association between CVC and hemorrhagic events, clinical data from day 1 to day 30 were collected. Intracranial hemorrhage had to be confirmed by computed tomography. Hematochezia required the presence of melena or hematemesis. Hematuria required the presence of gross hematuria. Pulmonary hemorrhage required the symptoms of coughing up blood. Hemorrhagic death was defined as death caused by hemorrhage. Catheter-related thrombosis (CRT), also referred to as symptomatic thrombosis, was defined as thrombosis in the vein in which the catheter was inserted and documented by screening ultrasound. An ultrasound examination for CRT was only performed in patients with CRT symptoms (pain, redness, swelling on CVC insertion area, limb edema, and dysfunction of CVC). Patients without CRT symptoms did not receive ultrasound examination for CRT. Catheter occlusion was defined as inability to withdraw blood or sluggish blood return, sluggish flow, resistance or inability to flush lumen, and inability to infuse fluid.

### Thirty-day survival rates

To verify an association between CVC and early death, data from day 0 to day 30 were collected. Early death was defined as deaths attributable to any cause between the first day of visit and the following 30 days.

### Statistical analysis

Statistical analyses were performed using SPSS 26.0. Categorical variables are presented as frequencies and were compared using the x^2^ test among the groups. If more than 20% of cells had less than the expected frequency of 5, Fisher’s exact test was alternatively used for analysis. Continuous variables are presented as the median (interquartile range, IQR) or mean and standard deviation and were compared using the Mann–Whitney U test and two independent samples t test for skewed data and normally distributed data, respectively. Thirty-day survival rates were analyzed using the Kaplan−Meier method and the log-rank test. A two-sided p value of ≤0.05 was considered statistically significant.

## Results

### Baseline characteristics

Ninety-five patients (48 females and 47 males) were included in our study. Of the total of 95 patients, 39 (41%) patients in the CVC group (36 patients with a peripherally inserted central catheter, 1 patient with an internal jugular vein catheter, 2 patients with subclavian vein catheters) and 56 (59%) patients in the non-CVC group were included in this retrospective study. Thirty-six (37.9%) patients, 28 (29.5%) and 31 (32.6%) were regarded as high-risk, intermediate-risk and low-risk individuals, respectively. Twenty-eight (29.5%) patients, 51 (53.7%) patients, 7 patients (7.3%) and 9 patients (9.5%) received ATRA with ATO, ATRA with chemotherapy, ATRA plus ATO with chemotherapy, and ATRA monotherapy, respectively. ATRA was initiated ≤2 days in 85 (89%) patients. The median WBC, platelet count, PT, APTT, Fbg and D-dimer were 3.12 × 10^9^/L, 20 × 10^9^/L, 13 seconds, 27.1 seconds and 1.3 g/L, 5445.0 μg/L FEU, respectively. The median percentages of promyelocytes in the bone marrow (BM) and peripheral blood (PB) were 80% and 48%, and the median percentages of blasts in BM and PB were 3% and 0%, respectively. Overall, there were no significant differences in any of the included 95 patients between the CVC group and non-CVC group regarding sex, age >60 years, risk stratification, median WBC, platelet count, PT, APTT, Fbg, D-dimer, DIC score and timing of ATRA initiation ≤2 days (*p* = 0.90, 0.31, 0.12, 0.08, 0.30, 0.99, 0.71, 0.72, 0.23, 0.52 and 0.19, respectively), except for the induction regimens (*p* = 0.03). These patient characteristics are summarized in **Table 1**. Moreover, when we compared the difference in the dynamic trend of coagulation data between the CVC and non-CVC groups, 13 early death cases were excluded to minimize interference as much as possible. The remaining 82 cases (36 in the CVC group and 46 in the non-CVC group) were incorporated into the following research. As summarized in **Table 2**, the baseline characteristics mentioned above were also not significantly different between the two groups except for the induction regimens (*p* = 0.01). The median day of CVC insertion was 4 (IQR: 3, 5).

**Table 1:** Baseline characteristics of the 95 patients.

Characteristics	Total (n = 95)	CVC group (n = 39)	Non-CVC group (n = 56)	*P*
Gender, n (%)				0.90
Male	47 (49.5)	19 (48.7)	28 (50.0)	
Female	48 (50.5)	20 (51.3)	28 (50.0)	
Age, n (%)				0.31
>60	13 (13.7)	7 (17.9)	6 (10.7)	
≤60	82 (86.3)	32 (82.1)	50 (89.3)	
Risk stratification, n (%)				0.12
High	36 (37.9)	10 (25.6)	26 (46.4)	
Intermediate	28 (29.5)	14(35.9)	14 (25.0)	
Low	31 (32.6)	15 (38.5)	16 (28.6)	
WBC, × 10^9^/L	3.12 (1.37, 17.58)	2.36 (1.01, 11.14)	4.17 (1.39, 25.70)	0.08
Hb, g/L	81.5 ± 25.7	83.6 ± 24.9	79.9 ± 26.4	0.50
PLT, × 10^9^/L	20.0 (12.0, 38.0)	22.0 (13.0, 43.0)	17.00(11.2, 34.3)	0.30
D-Dimer^a^, μg/L FEU	5445.0 (3432.5, 12725.0)	7310.0 (3646.5, 10000.0)	4480.0 (2895.0, 13850.0)	0.23
PT, s	13.0 (12.2, 15.0)	12.9 (12.4, 14.5)	13.2 (12.0, 15.7)	0.99
APTT, s	27.1 (24.6, 30.7)	26.9 (24.9, 30.8)	27.1 (24.3, 30.6)	0.71
Fbg, g/L	1.3 (0.9, 2.2)	1.3 (0.9, 2.2)	1.3 (1.0, 2.2)	0.72
Promyelocytes of bone marrow, %	80.0 (70.5, 89.5)	77.5 (70.5, 87.0)	80.3 (71.5, 89.5)	0.52
Blasts of marrow, %	3 (0.1, 8.5)	3.5 (01.0, 9.5)	2.8 (0.5, 7.4)	0.44
Promyelocytes of blood, %	48.0 (18.0, 76.0)	36.0 (12.0, 65.0)	54.0 (22.3, 80.9)	0.06
Blasts of blood, %	0.0 (0.0, 3.0)	0.0 (0.0, 5.0)	1.0 (0.0, 2.0)	0.98
Induction regimen, n (%)				0.03
ATRA + ATO	28 (29.5)	6 (15.4)	22 (39.3)	
ATRA + chemotherapy	51 (53.7)	28 (71.8)	23 (41.1)	
ATRA +ATO + chemotherapy	7 (7.4)	2 (5.1)	5 (8.9)	
ATRA only	9 (9.5)	3 (7.7)	6 (10.7)	
DIC **^a^** score, n (%)				0.52
<5	55 (40.9)	21 (55.3)	34 (61.8)	
≥5	38 (59.1)	17 (44.7)	21 (38.2)	
Timing of ATRA initiation ≤2 days, n (%)				0.19
Yes	85 (89.5)	37 (94.9)	37 (94.9)	
No	10 (10.5)	2 (5.1)	2 (5.1)	

^a^ One patient in the CVC group and one patient in the non-CVC group did not have a record of D-dimer at admission.

**Table 2:** Baseline characteristics of the 82 patients who survived the first 30 days.

Characteristics	Total (n = 82)	CVC (n = 36)	Non-CVC (n = 46)	*P*
Gender, n (%)				0.69
Male	39 (47.6)	18 (50.0)	21 (45.7)	
Female	43 (52.4)	18 (50.0)	25 (54.3)	
Age, n (%)				0.29
>60	8 (8.0)	5 (3.5)	3 (4.5)	
≤60	74 (74.0)	31 (32.5)	43 (41.5)	
Risk stratification, n (%)				0.39
High	27 (32.9)	9 (25.0)	18 (39.1)	
Intermediate	25 (30.5)	12 (33.3)	13 (28.3)	
Low	30 (36.6)	15 (41.7)	15 (32.6)	
WBC, × 10^9^/L	2.8 (1.4, 15.3)	2.5 (1.0, 10.8)	3.1 (1.4, 22.7)	0.28
PLT, × 10^9^/L	18.5 (11.0, 41.3)	21.5 (11.5, 44.5)	17.0 (11.0, 35.8)	0.38
Hb, g/L	80.3 ± 24.9	82.4 ± 24.9	78.6 ± 24.9	0.50
D-Dimer, μg/L FEU^a^	5500.0 (3430.0, 10000.0)	7580.0 (3700.0, 10000.0)	4328.5 (2805.0, 11380.0)	0.15
PT, s	12.9 (12.1, 14.8)	12.8 (12.3, 13.9)	13.1 (11.9, 15.3)	0.89
APTT, s	27.9 ± 4.2	27.8 ± 4.3	28.1 ± 4.2	0.72
Fbg, g/L	1.3 (0.9, 2.3)	1.4 (0.9, 2.2)	1.3 (0.9, 2.4)	0.96
Promyelocytes of blood, %	47.5 (17.8, 71.3)	38.5 (13.3, 65.8)	50.5 (21.3, 78.3)	0.30
Promyelocytes of bone marrow, %	79.8 (70.5, 89.5)	79.5 (70.8, 88.9)	79.8 (70.0, 89.5)	0.91
Blasts of blood, %	0.0 (0.0, 2.0)	0.0 (0.0, 4.8)	0.5 (0.0, 2.0)	0.77
Blasts of marrow, %	2.8 (0.9, 8.8)	3.0 (1.0, 9.3)	2.8 (0.5, 7.8)	0.65
Induction regimen, n (%)				0.01
ATRA + ATO	26 (31.7)	6 (16.7)	20 (43.5)	
ATRA + chemotherapy	49 (59.8)	28 (77.8)	21 (45.7)	
ATRA + ATO + chemotherapy	7 (8.5)	2 (5.6)	5 (10.9)	
ATRA only	0 (0)	0 (0)	0 (0)	
DIC **^b^** score, n (%)				0.51
<5	49 (61.3)	20 (57.1)	29 (64.4)	
≥5	31 (38.8)	15 (42.9)	16 (35.6)	
Timing of ATRA initiation ≤ day 2, n (%)				0.29
Yes	73 (89.0)	34 (94.4)	39 (84.8)	
No	9 (11.0)	2 (5.6)	7 (15.2)	
Timing of CVC, median (IQR)	NA	4 (3, 5)	NA	**NA**

^a, b,^ One patient in the CVC group and one patient in the non-CVC group did not have a record of D-dimer at admission; NA, not applicable.

### CVC insertion and use

Of the 39 patients who inserted CVCs, 36 patients inserted a PICC and 3 patients inserted a CICC (1 patient with an internal jugular vein catheter, 2 patients with subclavian vein catheters). No patients received tunneled catheters. The reasons for CVC insertion were mainly as follows: chemotherapy (92%), monitoring hemodynamics (8%), blood transfusion (100%) and intravenous infusion (100%). In the PICC group, PICCs were inserted through the basilic vein in 64% of cases and all catheters inserted were 4 Fr catheters. In the CICC group, CICCs were inserted through the subclavian vein in 67% of patients and all CICCs inserted were 7 Fr catheters. Sixteen (44%) patients in the PICC group and 3 (100%) patients in the CICC group were inserted in the right arm. The characteristics and use of the catheters in both groups are summarized in **Table 3**.

**Table 3:** Characteristics of CVCs and their use.

Characteristic	Total CVC (n = 39)	PICC group (n = 36)	CICC group (n = 3)
CVC insertion site, n (%)			
Basilic vein	23 (59)	23 (64)	NA
Cephalic vein	5 (13)	5 (14)	NA
Median cubital vein	8 (21)	8 (22)	NA
Subclavian vein	2 (5)	NA	2 (67)
Internal jugular vein	1 (2)	NA	1 (33)
Right side	19 (49)	16 (44)	3 (100)
Left side	20 (51)	20 (56)	0 (0)
CVC type, n (%)			
Non-tunneled	39 (100)	36 (100)	3 (100)
4 Fr	36 (92)	36 (100)	NA
5 Fr	0 (0)	0 (0)	NA
6 Fr	0 (0)	0 (0)	NA
7 Fr	3 (8)	0 (0)	3 (100)
Single lumen	36 (92)	36 (100)	0 (0)
Double lumen	3 (8)	0 (0)	3 (100)
Reasons for CVC			
Chemotherapy	36 (92)	36 (100)	0 (0)
Hemodynamic monitoring	3 (8)	0 (0)	3 (100)
Intravenous infusion	39 (100)	36 (100)	3 (100)
Blood transfusion	39 (100)	36 (100)	3 (100)

NA, not applicable.

### Dynamic trend of coagulation data

**Figure 1:**
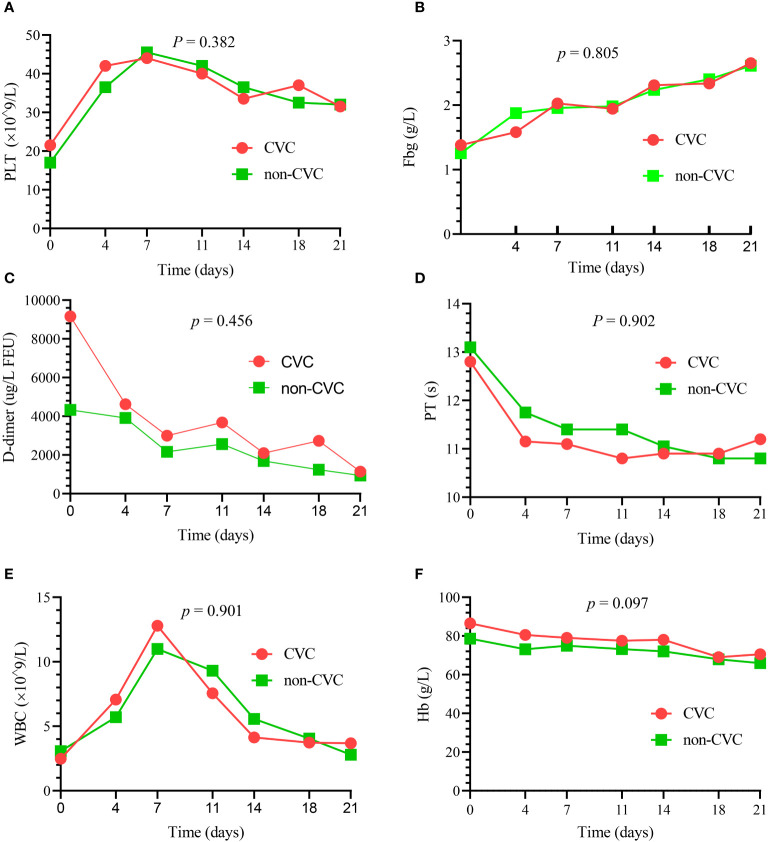
The dynamic changes in the principal blood parameters in 82 patients who survived the first 30 days. There were no significant differences in the median platelet (PLT) **(A)**, median Fbg **(B)**, median D-dimer **(C)**, median PT **(D)**, median WBC **(E)** and median Hb **(F)** between the CVC and non-CVC groups at seven time points (*p* = 0.382, 0.805, 0.456, 0.902, 0.901 and 0.097, respectively).

There were no significant differences in the dynamic trend of coagulation data in the 82 patients who survived the first 30 days between the CVC and non-CVC groups. The median platelet, PT, Fbg, D-dimer, WBC and Hb values at each time point (day 0, day 4, day 7, day 11, day 14, day 18 and day 21) during induction therapy showed no significant difference (*p* = 0.382, 0.902, 0.805, 0.456, 0.901 and 0.097, respectively), as shown in **Figure 1**. In addition, as summarized in **Table 4**, the amounts of platelets and Fbg used were also not significantly different between the two groups. The median transfusion amount of platelets was 5 units (IQR: 3.0, 7.8) in patients with CVCs and 4.5 units (IQR: 2.0, 7.0) in the non-CVC group (*p* = 0.34). At the same time, the median transfusion amount of human Fbg was 6.8 g (IQR: 3.0, 12.3) in the CVC group and 6.0 g (IQR: 2.0, 9.0) in the non-CVC group (*p* = 0.36).

**Table 4:** Summary of blood transfusion in the 82 patients who survived the first 30 days.

Characteristics	Total (n = 82)	CVC (n = 36)	Non-CVC (n = 46)	*P*
PLT infused (units)	5.0 (2.8, 7.0)	5.0 (3.0, 7.8)	4.5 (2.0, 7.0)	0.34
Fbg infused (g)	6.3 (2.5, 10.5)	6.8 (3.0, 12.3)	6.0 (2.0, 9.0)	0.36
Last day of PLT transfusion	20.0 (16.0, 24.0)	21.0 (18.0, 24.8)	19.0 (8.5, 23.3)	0.24
Last day of Fbg transfusion	8.0 (2.8, 14.0)	8.5 (3.0, 15.5)	7.5 (2.0, 14.0)	0.68

To avoid the interference of transfused component blood, we provided the last day of platelet and Fbg transfusion as more convincing evidence, indicating the time of platelet recovery and coagulopathy correction. As summarized in **Table 4**, the time of platelet recovery and coagulopathy correction were not significantly different. Although the median time of platelet recovery in the CVC group was longer than that in the non-CVC group (21 vs. 19, respectively), it did not differ statistically (*p* = 0.24). Meanwhile, coagulopathy correction (last day of Fbg transfusion) was faster in the non-CVC group than in the CVC group (7.5 vs. 8.5, respectively), but these differences were not statistically significant (*p* = 0.68).

### Hemorrhagic events

Overall, 28 (29.5%) of the 95 patients developed hemorrhagic events, and 9 (9.5%) patients developed hemorrhagic deaths. The incidences of total bleeding events and hemorrhagic deaths during induction therapy were lower in the CVC group than in the non-CVC group (7 (17.9%) vs. 21 (37.5%), *p* = 0.04; 0 (0.0%) vs. 9 (16.1%), *p* = 0.01). Specifically, 5 (12.8%) patients in the CVC group and 9 (16.1%) patients in the non-CVC group had oral, eye or nasal bleeding (*p* = 0.66), 2 (5.1%) patients in the CVC group and 4 (7.1%) patients in the non-CVC group experienced pulmonary hemorrhage (*p* = 1.00), and 1 (2.6%) patient in the CVC group and 8 (14.3%) patients in the non-CVC group experienced intracranial hemorrhage. Catheter occlusion occurred in 5 cases while no symptomatic catheter-related thrombosis was observed. These data are summarized in **Table 5**.

**Table 5:** Summary of hemorrhagic and thrombotic events in all 95 patients.

Characteristics	Total (n = 95)	CVC (n = 39)	Non-CVC (n = 56)	*P*
Hemorrhagic events total, n (%)				0.04
Yes	28 (29.5)	7 (17.9)	21 (37.5)	
No	67 (70.5)	32 (82.1)	35 (62.5)	
Hemorrhagic deaths, n (%)				0.01
Yes	9 (9.5)	0 (0.0)	9 (16.1)	
No	86 (90.5)	39 (100.0)	47 (83.9)	
Epistaxis, gingival bleeding and fundus hemorrhage, n (%)				0.66
Yes	14(14.7)	5 (12.8)	9 (16.1)	
No	81 (85.3)	34 (87.2)	47 (83.9)	
Intracranial hemorrhage, n (%)				0.08
Yes	9 (9.5)	1 (2.6)	8 (14.3)	
No	86 (90.5)	38 (97.4)	48 (85.7)	
Hematuresis/hematochezia, n (%)				1.00
Yes	0 (0)	0 (0)	0 (0)	
No	95 (100)	39 (100)	47 (100)	
Pulmonary hemorrhage, n (%)				1.00
Yes	6 (6.3)	2 (5.1)	4 (7.1)	
No	89 (93.7)	37 (94.9)	52 (92.9)	
Early deaths, n (%)				0.16
Yes	13 (13.7)	3 (7.7)	10 (17.9)	
No	82 (86.3)	36 (92.3)	46 (82.1)	
Catheter-related thrombosis, n (%)				NA
Yes	NA	0 (0)	NA	
No	NA	39 (100)	NA	
Catheter occlusion, n (%)				NA
Yes	NA	5 (13)	NA	
No	NA	34 (87)	NA	

NA, not applicable.

### Thirty-day survival analysis

**Figure 2:**
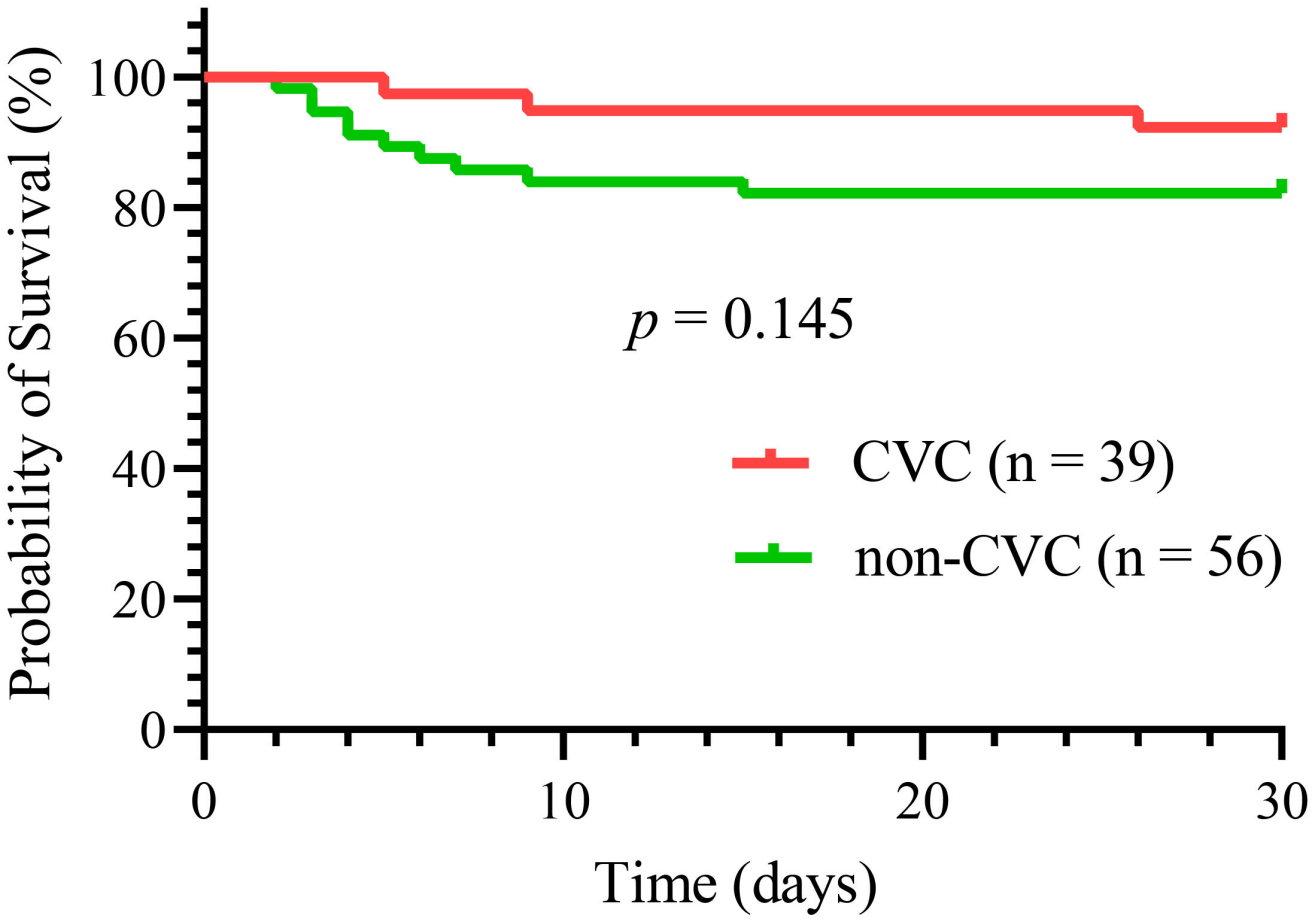
30-day survival analysis for the CVC and non-CVC groups in all 95 patients.

Overall, 13 (13.7%) early deaths occurred in all 95 patients in our study. Of all the early deaths, hemorrhage was the most common cause of induction death (9 patients, 69%), followed by respiratory failure (2 patients, 15%), differentiation syndrome (1 patient, 8%) and multiorgan failure, mostly likely resulting from thrombotic thrombocytopenic purpura (1 patient, 8%). Hemorrhagic mortalities were almost exclusively caused by intracranial (8 patients, 8.4%) and pulmonary hemorrhages (1 patient, 1.1%). Thirty-day survival did not differ significantly between the two groups, as shown in **Figure 2** (*p* = 0.15). Three deaths (7.7%) occurred in the CVC group compared with ten (17.9%) in the non-CVC group. The causes of early death in the CVC group were one differentiation syndrome, one multiorgan failure, and one aspiratory failure. In the 10 patients who died in the non-CVC group at 30 days, the causes of death were eight intracranial hemorrhages, one pulmonary hemorrhage and one aspiratory failure.

## Discussion

In this manuscript, we explored the safety of CVCs in APL patients during induction therapy. The results suggested that coagulopathies in the CVC group could be corrected as rapidly as that in the non-CVC group. Meanwhile, CVCs do not increase the risk of bleeding events, and the 30-day survival rate for the CVC group was comparable to that for the non-CVC group.

Our study showed that the resolution of coagulopathies was not significantly different between the CVC and non-CVC groups at different points during days 0–21 for the 82 patients surviving more than 30 days, as shown in **Table 4**; **Figure 1**. The median Fbg recovery was 8 days in our study, and this result was in agreement with the outcome of other studies (13), which suggested that Fbg was reversed within two weeks in most of the patients. Our findings also support the view that Fbg is an early and sensitive indicator of improvement in coagulopathy (15). A recent study showed that Fbg <1.0 g/L was an independent risk factor for early death (16). Therefore, the recovery of Fbg is essential for the prevention of early death. Interestingly, the recovery and consumption of Fbg were not significantly different between the CVC group and the non-CVC group. These results suggest that CVCs do not worsen the pace of Fbg recovery and increase the consumption of Fbg.

Hemorrhage is the common complications during induction therapy for APL patients. In our study, 28 (29.5%) patients developed hemorrhagic events, and 9 (9.5%) patients developed hemorrhagic deaths. The hemorrhagic death rates in our study were higher than those in both the clinical studies of LPA96 and LPA99 (5.1% and 5%, respectively) (17). These differences may result from the inclusion of patients who were not fit or too old for inclusion in clinical trials. Moreover, hemorrhagic deaths were exclusively caused by intracranial (8 patients, 88.9%) and pulmonary hemorrhagic events (1 patient, 11.1%) in our study, and these results are consistent with what de la Serna et al. reported (17) Surprisingly, the incidence of intracranial hemorrhage in the CVC group was lower than that in the non-CVC group, and the difference significantly differed (0.0% vs. 16.1%, respectively, *p* = 0.01). These results suggest that CVCs do not increase the risk of hemorrhagic events, especially the most common intracranial hemorrhage.

Although a significant improvement has been reported in terms of the rates of early death, there are still discrepancies regarding the early death rates among clinical, registration, or retrospective studies. In our study, the early death rate was 13.7% for all 95 included patients within the first 30 days, as shown in **Table 5**; **Figure 2**. These results are higher than a study that revealed an overall induction mortality rate of 3.2% in APL patients treated with ATRA, idarubicin, and IV arsenic trioxide (18) but lower than that in a Swedish registry study (29%) (19). The main reason for the higher early death rate in our study compared with clinical trials is probably the inclusion of patients with very early deaths, as well as of patients who were not fit or too old for inclusion in clinical trials. The PETHEMA group reported that approximately 5% of patients were considered not eligible for induction therapy due to very poor clinical conditions, mostly due to lethal or life-threatening hemorrhagic events before starting therapy (20). In addition, the early death rate was lower in the CVC group than in the non-CVC group (7.7% vs. 17.9%, respectively); however, this difference was not significant (*p* = 0.16). Early death was closely linked to age >60 years (17) and several blood chemistry characteristics, such as initial Fbg level and WBC (21). In our study, these characteristics were comparable, and the early death rate was not significantly different between the two groups. Of note, the induction regimens, which may impact early death, were not similar between the two groups in our study. Studies of anthracycline-free regimens using arsenic trioxide (ATO) showed less early death and fewer reported cases of hemorrhagic death (22, 23). While more patients used the arsenic trioxide (ATO)-containing regimens in the non-CVC group in our study, the early death rate was not significantly different between the two groups. Overall, these results suggest that CVCs do not increase the risk of early death.

However, our study has some limitations. First, although the risk stratification in both groups were comparable (*p* = 0.12), the percentage of patients with high risk was lower in the CVC group than in the non-CVC group (10% vs. 26%). Moreover, APL patients with a higher risk had a greater incidence of hemorrhage than did patients with an intermediate/low risk during induction therapy (16, 19). Therefore, due to the retrospective nature and the relatively small size of our study, this issue might still be an important confounding variable and further prospective studies are needed. Second, the reasons why some patients in the non-CVC group did not have CVCs placed were unclear because they depended entirely on the clinician’s determination. Third, many factors that could interfere with the hemostatic system in APL, such as concomitant infections, were not analyzed.

In conclusion, our study suggested that CVCs did not increase the hemorrhagic risk in APL patients during induction therapy, and we offered information that a CVC should be considered in a clinical situation.

## Data availability statement

The original contributions presented in the study are included in the article/supplementary material. Further inquiries can be directed to the corresponding authors.

## Ethics statement

The studies involving humans were approved by the First Affiliated Hospital of Shantou University Medical College Ethics Committee. The studies were conducted in accordance with the local legislation and institutional requirements. Written informed consent for participation was not required from the participants or the participants’ legal guardians/next of kin due to the retrospective nature of the study.

## Author contributions

MC: Data curation, Formal Analysis, Methodology, Writing – original draft, Writing – review & editing. JH: Data curation, Investigation, Writing – original draft. DZ: Data curation, Formal Analysis, Investigation, Supervision, Writing – original draft, Writing – review & editing. FC: Data curation, Formal Analysis, Investigation, Supervision, Writing – original draft, Writing – review & editing. YS: Data curation, Formal Analysis, Investigation, Supervision, Writing – original draft, Writing – review & editing.
